# miRNA-Processing Gene Variants and Neuroblastoma Susceptibility: A Pilot Case-Control Study

**DOI:** 10.7759/cureus.106920

**Published:** 2026-04-12

**Authors:** Ioannis Kyriakidis, Iordanis Pelagiadis, Georgia Martimianaki, Maria Stratigaki, Nikolaos Katzilakis, Eftichia Stiakaki

**Affiliations:** 1 Department of Pediatric Hematology-Oncology and Autologous Hematopoietic Stem Cell Transplantation Unit, University Hospital of Heraklion and Laboratory of Blood Diseases and Childhood Cancer Biology, School of Medicine, University of Crete, Heraklion, GRC

**Keywords:** argonaute, dicer, drosha, genetic susceptibility, microrna, neuroblastoma, polymorphism

## Abstract

Background: Neuroblastoma (NB) is the most common extracranial solid tumor in children, but the inherited factors influencing susceptibility are not fully understood. Given the link between defective microRNA biogenesis and aggressive NB, germline variants in key miRNA-processing genes are plausible candidate modifiers. This pilot case-control study aimed to assess whether five selected SNPs in *DICER1*, *DROSHA*, and *AGO1* are associated with NB susceptibility in a Greek pediatric population.

Materials and methods: This exploratory case-control study included 200 children from Crete, Greece, comprising 25 with NB and 175 healthy controls recruited during routine health visits. Five SNPs in *DICER1*, *DROSHA*, and *AGO1* genes were genotyped using TaqMan assays. Logistic regression analyses were adjusted for age and sex, with multiple testing corrections applied via Bonferroni and false discovery rate (FDR) methods. Due to the limited number of NB cases, analyses within this group were considered exploratory.

Results: In the adjusted primary analyses, *DROSHA* rs3805500 demonstrated the strongest association with NB susceptibility (per G allele OR 3.43, 95% CI 1.69-6.94) and remained significant after Bonferroni and FDR corrections. *DROSHA* rs642321 showed a nominal association that did not retain significance after correction for multiple testing, while *DICER1* rs3742330 exhibited a borderline association in the adjusted model. No significant associations were observed for *AGO1* rs636832 or *DROSHA* rs10035440. Exploratory within-case analyses provided limited descriptive insights and require validation in larger cohorts.

Conclusions: This pilot study suggests that genetic variations in the miRNA-processing pathway may affect NB susceptibility. Further research involving larger, ethnically diverse cohorts, along with expanded genotype-expression analyses and functional validation, is essential before clinical or prognostic applications can be established.

## Introduction

Neuroblastoma (NB), although rare with an incidence of 10-12 cases per million children, is the most common extracranial solid tumor in children and accounts for approximately 15% of pediatric cancer deaths [1]. NB exhibits significant clinical variability, from spontaneous regression to aggressive metastasis. Markers of aggressive biology include *MYCN* amplification, gain-of-function *ALK* mutations, and aberrations in the RAS pathway or TP53 [2]. Familial NB is rare (<2%), while susceptibility to sporadic cases is partly polygenic, with about 20% potentially identifiable by risk scores based on variants at loci such as 6p22 (*CASC15/NBAT1*), *BARD1*, *LMO1*, 6q16 (*HACE1* and *LIN28B*, regulators of the let-7 microRNA family), and *MMP20* [3,4].

MicroRNAs are short non-coding RNAs that regulate gene expression post-transcriptionally. Their maturation depends on tightly coordinated processing by the Drosha-DGCR8 microprocessor complex, nuclear export machinery, Dicer, and the Argonaute-containing RNA-induced silencing complex. Disruption of this pathway can alter differentiation, proliferation, apoptosis, and treatment response in cancer, including NB [5,6]. MicroRNA dysregulation is closely linked to NB tumorigenesis, progression, treatment resistance, and prognosis [7,8]. Key alterations involve tumor-suppressive miR-34a, miR-204, and let-7 family members, as well as the oncogenic miR-17~92 cluster and ALK-regulated miRNAs [3,9-11]. Accordingly, knockdown or silencing of either *DICER1* or *DROSHA* promoted the growth of NB cell lines. Low Dicer or Drosha expression has been associated with global miRNA downregulation, high-risk neuroblastoma, and poor outcomes, which makes germline variation in this pathway biologically relevant [12].

Single-nucleotide polymorphisms (SNPs) in miRNA biogenesis genes have been linked to susceptibility to various cancers [13]. In NB, however, germline studies have mainly focused on genome-wide susceptibility loci or variants within individual miRNAs rather than on the canonical miRNA-processing machinery [14,15]. This pilot case-control study aimed to evaluate whether five selected single-nucleotide polymorphisms in the miRNA-processing genes *DICER1*, *DROSHA*, and *AGO1* are associated with NB susceptibility in a Greek pediatric population. Specifically, we examined *DICER1* rs3742330, *AGO1* rs636832, and *DROSHA* rs642321, rs3805500, and rs10035440. Given the rarity of NB and the small sample size, all analyses were considered exploratory.

## Materials and methods

Study population

The current retrospective case-control study enrolled 200 participants: 25 children diagnosed and treated for NB at the tertiary Pediatric Hematology-Oncology Department of the University Hospital of Heraklion and 175 unrelated healthy controls from the same geographic region. Peripheral blood samples from patients were obtained during remission or at a later disease phase, with no detectable circulating neuroblasts in children with prior marrow involvement. The median follow-up of the NB cohort was 10.3 years (interquartile range (IQR): 17.1 years).

Controls were recruited during routine checkups. Eligibility required normal growth, no chronic medical conditions, no regular medication use, no previous hospitalization, and no personal or family history of cancer. Cases and controls were not age-matched; therefore, age and sex were included as covariates in all association models. Clinical variables collected for the NB cohort included stage, *MYCN* amplification, high-risk classification, relapse, and survival status.

Written informed consent was obtained from legal guardians and adolescents when appropriate. The study was conducted in accordance with the Declaration of Helsinki and was approved by the Institutional Review Board of the University General Hospital of Heraklion (approval number: 1732/01 Nov 2024; Scientific Council 30542-30550/18 Oct 2024).

Genotyping

Genomic DNA was extracted from peripheral blood using the PureLink Genomic DNA Mini Kit (Invitrogen, Thermo Fisher Scientific, Waltham, Massachusetts, United States). The five SNPs were genotyped using predesigned TaqMan SNP Genotyping Assays (Thermo Fisher Scientific, Waltham, Massachusetts, United States) on a Bio-Rad CFX96 Real-Time PCR Detection System (Hercules, California, United States), following the manufacturer's instructions: *DICER1* rs3742330 (C__27475447_10), *AGO1* rs636832 (C___1079151_10), and *DROSHA* rs642321 (C___3197426_20), rs3805500 (C___3197387_20), and rs10035440 (C__11277968_10) (Applied Biosystems, Thermo Fisher Scientific Inc., Carlsbad, California, United States) [16,17]. The genotyping call rate was 100%, and duplicate samples showed 100% concordance.

Statistical analysis

Hardy-Weinberg equilibrium was assessed in controls with the chi-squared test. Continuous variables were summarized as medians with IQRs and categorical variables as counts with percentages. Age and sex distributions were compared between groups using the Mann-Whitney U test and Fisher's exact test, respectively. Logistic regression adjusted for age and sex was used to estimate odds ratios (ORs) and 95% confidence intervals (CIs) for susceptibility analyses. To limit model multiplicity, the prespecified primary analysis emphasized the log-additive model; alternative inheritance models identified by SNPStats were considered supportive and descriptive [18]. Bonferroni and false discovery rate (FDR) corrections were applied across the primary susceptibility analyses. Because the outcome analysis included very few relapse and death events, those within-case comparisons were treated as exploratory and hypothesis-generating only. Analyses were performed with IBM SPSS Statistics for Windows, Version 29.0.2.0 (IBM Corp., Armonk, New York, United States) and SNPStats.

Post hoc design calculations indicated limited power to detect modest effects: under a log-additive rare-disease approximation, power to detect an OR of 2.0 ranged from 28% to 62% across the three associated loci, and 80% power would have required approximately 77-203 cases with seven controls per case.

## Results

All five polymorphisms were in Hardy-Weinberg equilibrium among controls. Controls were older than cases (p<0.001), consistent with the early age distribution of NB, whereas sex distribution did not differ significantly between groups (p=0.135). Accordingly, age and sex were retained in all regression models (Table 1).

**Table 1:** Participant characteristics Data are presented as median (IQR) or n (%). Two-sided p<0.05 was considered statistically significant. *Mann-Whitney U test; U=873.0. **Fisher's exact test, two-sided. NB: neuroblastoma; INSS: International Neuroblastoma Staging System

Variable	NB cases (n=25)	Controls (n=175)	P-value
Age at sampling, median (IQR), years	2.26 (2.78)	4.90 (9.00)	<0.001*
Female sex, n (%)	8 (32)	86 (49.1)	0.135**
Male sex, n (%)	17 (68)	89 (50.9)	
INSS stage 1, n (%)	3 (12)	-	-
INSS stage 2A/2B, n (%)	6 (24)	-	-
INSS stage 3, n (%)	0 (0)	-	-
INSS stage 4, n (%)	13 (52)	-	-
INSS stage 4S, n (%)	3 (12)	-	-
MYCN amplification, n (%)	7 (28)	-	-
High-risk classification, n (%)	13 (52)	-	-
Relapse, n (%)	8 (32)	-	-
Alive at the last follow-up, n (%)	17 (68)	-	-

Genotype distributions are summarized in Table 2, and the primary susceptibility analyses are shown in Table 3. In the adjusted primary analyses, *DROSHA* rs3805500 showed the most robust association with NB susceptibility: the G allele was associated with increased risk, and this association remained significant after both Bonferroni and FDR corrections. *DROSHA* rs642321 showed a nominal association but was not retained after multiplicity correction and should be interpreted with caution due to sparse genotype strata. *DICER1* rs3742330 showed a borderline association in the adjusted model, whereas *AGO1* rs636832 and *DROSHA* rs10035440 showed no significant associations.

**Table 2:** Genotype distributions of the studied polymorphisms Data are presented as n (%). SNP: single-nucleotide polymorphism

Gene/SNP	Genotype	Cases, n (%)	Controls, n (%)
*DICER1 *rs3742330	AA	14 (56)	147 (84)
	AG	11 (44)	26 (14.9)
	GG	0 (0)	2 (1.1)
*AGO1 *rs636832	GG	18 (72)	134 (76.6)
	AG	7 (28)	39 (22.3)
	AA	0 (0)	2 (1.1)
*DROSHA *rs642321	CC	24 (96)	106 (60.6)
	CT	1 (4)	65 (37.1)
	TT	0 (0)	4 (2.3)
*DROSHA *rs3805500	AA	7 (28)	81 (46.3)
	AG	4 (16)	76 (43.4)
	GG	14 (56)	18 (10.3)
*DROSHA *rs10035440	TT	16 (64)	110 (62.9)
	CT	8 (32)	54 (30.8)
	CC	1 (4)	11 (6.3)

**Table 3:** Primary susceptibility analyses under the log-additive model Data are presented as OR (95% CI). ORs were estimated by binary logistic regression under a log-additive genetic model adjusted for age and sex. The numerical test statistic shown is the Wald chi-square. Two-sided p<0.05 was considered statistically significant. Bonferroni-corrected and Benjamini-Hochberg FDR-adjusted p-values were calculated across the five primary susceptibility analyses. SNP: single-nucleotide polymorphism; FDR: false discovery rate

Gene/SNP	Additive model	Adjusted OR (95% CI)	Wald χ^2^	Nominal p	Bonferroni	FDR	Interpretation after multiplicity control
*DICER1 *rs3742330	Per G allele	2.62 (0.95-7.25)	3.47	0.063	0.315	0.105	No susceptibility signal detected
*AGO1 *rs636832	Per A allele	1.59 (0.54-4.74)	0.70	0.402	1.000	0.443	No susceptibility signal detected
*DROSHA *rs642321	Per T allele	0.09 (0.01-0.73)	5.09	0.024	0.120	0.060	Not retained after correction; effect size likely inflated by sparse cells
*DROSHA *rs3805500	Per G allele	3.43 (1.69-6.94)	11.72	<0.001	0.003	0.003	Most robust association; retained after Bonferroni and FDR
*DROSHA *rs10035440	Per C allele	0.73 (0.32-1.64)	0.59	0.443	1.000	0.443	No susceptibility signal detected

*MYCN* amplification was present in seven NB patients and was associated with a more advanced disease stage, but in this small series, it did not show a statistically significant association with relapse or survival.

Exploratory outcome analyses suggested that the *DICER1* rs3742330 AA and *AGO1* rs636832 AG genotypes might be associated with more favorable outcomes. Six patients carried the combined genotype pattern *DICER1* rs3742330 AA plus *AGO1* rs636832 AG, and all six were alive at the last follow-up. Because this subgroup was extremely small and no functional validation was available, these observations are reported as descriptive only and should not be interpreted as evidence of clinical utility.

## Discussion

This pilot case-control study suggests that germline variation in canonical miRNA-processing genes may be associated with NB susceptibility. Among the variants examined, *DROSHA* rs3805500 emerged as the strongest candidate signal. *DROSHA* rs642321 showed a nominal association that was not retained after multiplicity correction, while *DICER1* rs3742330 showed a borderline association in the adjusted primary model. These findings fit the broader concept that constitutional variation affecting miRNA biogenesis may modulate tumor susceptibility in parallel with established somatic drivers [7,8,10,12,13].

Biological plausibility supports the loci prioritized for further study, especially *DICER1* rs3742330 and *DROSHA* rs642321 and rs3805500. *DICER1* rs3742330 is a non-coding variant in the 3′-untranslated region that has been discussed as a regulator of transcript stability and miRNA-mediated control in other diseases, with minor allele G carriers displaying significantly lower Dicer mRNA expression [13,19,20]. *AGO1* rs636832 is an intronic variant, and prior work has linked Argonaute 1 expression to differentiation, proliferation, and apoptosis in NB models [13,21,22]. *DROSHA* rs642321 and rs3805500 are non-coding variants with predicted regulatory potential, and associations with these variants have been reported in other malignancies, including leukemias [17,23,24]. However, these mechanistic links remain inferential in the present study.

Public bioinformatic annotation may support regulatory plausibility (e.g., GTEx, HaploReg, or motif-prediction tools), but it does not establish functional relevance in NB and should be interpreted only as supportive context, as these resources rely largely on adult non-tumor data and are not substitutes for genotype-specific expression analyses in NB tissue or cell lines.

The apparent lack of association between *MYCN* amplification and outcome in this cohort should not be viewed as contradictory to the established NB literature [2,3]. More likely, it reflects limited statistical power: only seven patients had *MYCN* amplification, and the number of relapse and death events was small. In such settings, known prognostic factors can fail to reach significance simply because the sample is underpowered and clinically heterogeneous.

Several limitations should be acknowledged. First, the sample size was very small for a genetic association study, with some genotype groups containing only one or zero cases. This increases the risk of type 1 error, wide CIs, and inflated OR estimates, especially for rs642321. Second, the cohort was entirely from Crete, a geographically restricted population where founder effects or population-specific linkage disequilibrium might influence allele frequencies and cause the studied SNPs to tag local haplotypes rather than causal variants. Third, healthy controls were older than NB patients, which could introduce residual confounding despite adjusting for age. Fourth, we did not perform genotype-expression analyses in patient samples or conduct experimental functional validation. Overall, these issues suggest that the findings should be considered exploratory and hypothesis-generating.

The exploratory survival signal involving *DICER1* rs3742330 and *AGO1* rs636832 is intriguing, particularly because both genes participate in miRNA maturation and post-transcriptional silencing. Nevertheless, the subgroup consisted of only six children. A 100% survival observation in such a small subset cannot justify biomarker claims and should only motivate replication in larger cohorts with event-based time-to-outcome modeling. Accordingly, these outcome-related subgroup findings should be considered hypothesis-generating only and not interpreted as clinically actionable.

## Conclusions

This pilot study suggests that inherited variations in the miRNA-processing pathway may influence NB susceptibility, with *DROSHA* rs3805500 emerging as the most notable association within this dataset. However, these findings do not yet support the use of this marker in clinical practice. Further validation through larger, ethnically diverse cohorts, along with genotype-expression and functional analyses, is essential before considering any prognostic or therapeutic applications.
